# An atrioventricular node model incorporating autonomic tone

**DOI:** 10.3389/fphys.2022.976468

**Published:** 2022-09-15

**Authors:** Felix Plappert, Mikael Wallman, Mostafa Abdollahpur, Pyotr G. Platonov, Sten Östenson, Frida Sandberg

**Affiliations:** ^1^ Department of Biomedical Engineering, Lund University, Lund, Sweden; ^2^ Department of Systems and Data Analysis, Fraunhofer-Chalmers Centre, Gothenburg, Sweden; ^3^ Department of Cardiology, Clinical Sciences, Lund University, Lund, Sweden; ^4^ Department of Internal Medicine and Department of Clinical Physiology, Central Hospital Kristianstad, Kristianstad, Sweden

**Keywords:** atrial fibrillation, atrioventricular node, autonomic tone, tilt test, mathematical modeling, ECG, RR series characteristics, sample entropy

## Abstract

The response to atrial fibrillation (AF) treatment is differing widely among patients, and a better understanding of the factors that contribute to these differences is needed. One important factor may be differences in the autonomic nervous system (ANS) activity. The atrioventricular (AV) node plays an important role during AF in modulating heart rate. To study the effect of the ANS-induced activity on the AV nodal function in AF, mathematical modelling is a valuable tool. In this study, we present an extended AV node model that incorporates changes in autonomic tone. The extension was guided by a distribution-based sensitivity analysis and incorporates the ANS-induced changes in the refractoriness and conduction delay. Simulated RR series from the extended model driven by atrial impulse series obtained from clinical tilt test data were qualitatively evaluated against clinical RR series in terms of heart rate, RR series variability and RR series irregularity. The changes to the RR series characteristics during head-down tilt were replicated by a 10% decrease in conduction delay, while the changes during head-up tilt were replicated by a 5% decrease in the refractory period and a 10% decrease in the conduction delay. We demonstrate that the model extension is needed to replicate ANS-induced changes during tilt, indicating that the changes in RR series characteristics could not be explained by changes in atrial activity alone.

## 1 Introduction

Atrial fibrillation (AF) is the most common supraventricular tachyarrhythmia (Hindricks et al., 2020). Characteristic for AF is an increased and irregular atrial activity that results in a rapid and irregular ventricular activation. Atrial fibrillation is linked to substantial morbidity and mortality, and is a significant burden to patients, physicians, and healthcare systems globally. Two main strategies of AF treatments are rate control and rhythm control. Rate control is one of the corner stones of AF management, however the effect of individual rate-control drugs are difficult to predict in advance. This is why the choice of a rate-control drug today remains empiric and driven largely by their safety profile and contraindications rather than predicted efficacy. Therefore, the complex mechanisms of AF have to be better understood to personalize the treatment and reduce the burden of AF on the healthcare system.

It has been shown that the autonomic nervous system (ANS) is contributing to the initiation and maintenance of AF (Shen and Zipes, 2014). Either a predominance in sympathetic or in parasympathetic modulation has been observed to initiate an episode of paroxysmal atrial fibrillation (PAF); and in some patients, both the sympatho-vagal and vagal predominances have been observed to initiate PAF episodes (Lombardi et al., 2004). Hence, differences in the ANS activity among patients may be an important factor behind the inter-patient differences in response to treatment. To investigate the ANS-induced changes to the pathophysiology of AF, the effect of the ANS has to be quantified. One common method to quantify the autonomic tone during normal sinus rhythm (NSR) is by heart rate variability (HRV) (Sassi et al., 2015). In sinus rhythm, HRV can be used to obtain information about the function of the sinoatrial (SA) node. This information is valuable for the quantification of the autonomic tone, because the SA node is densely innervated by the ANS (Shen and Zipes, 2014; George et al., 2017). In AF, however, HRV cannot be used to quantify the autonomic tone, because the heart beats are not initiated in the SA node.

Instead, the ventricular rhythm during AF is determined by the atrial electrical activity and the subsequent AV nodal modulation. Since the AV node is densely innervated by the ANS, characterizing the AV nodal behavior during AF may give valuable information about the autonomic tone. Results from previous studies suggest that the heart rate, as well as the heart rate variability, quantified by RR rmssd, and heart rate irregularity, quantified by RR sample entropy, are affected by *β*-blocker induced changes in sympathetic response (Corino et al., 2015). We hypothesize that such changes in the heart rate and its variability and irregularity reflect ANS-induced changes in the AV node. The ANS-induced changes on the cardiac electrophysiology can be studied using head-up and head-down tilt test, which in a previous study was shown to affect electrophysiological properties of atrial myocardium during AF (Östenson et al., 2017). It is unclear if the changes in the heart rate and its variability and irregularity are explained by the changes in the atrial electrophysiology alone or also by changes in the AV nodal properties. Investigating how the ANS is modulating the heart rate during AF is a complex task and requires a model based analysis.

Previously, several AV node models have been proposed that incorporate important characteristics of the AV nodal structure and electrophysiology in their design. Characteristic for the AV node is its dual-pathway physiology enabling a parallel excitation propagation of impulses with different electrophysiological properties (George et al., 2017). For example, the slow pathway (SP) has a longer conduction delay and shorter refractory period compared to the fast pathway (FP) (George et al., 2017). Furthermore, the refractory period and conduction delay are dynamic and depend on the recent history of the conducted and blocked impulses in the AV nodal tissue (George et al., 2017; Billette and Tadros, 2019). Early models of the AV node did not account for the dual-pathway physiology (Cohen et al., 1983; Jørgensen et al., 2002; Rashidi and Khodarahmi, 2005; Mangin et al., 2005; Lian et al., 2006). Later models have incorporated this feature, represented by separate refractory periods (Corino et al., 2011; Henriksson et al., 2016; Inada et al., 2017; Wallman and Sandberg, 2018) and separate conduction delays (Climent et al., 2011b; Inada et al., 2017; Wallman and Sandberg, 2018). However, no models have explicitly incorporated ANS-induced changes in their model description.

Therefore, the aim of the present study is to incorporate ANS-induced changes into the AV node network model previously proposed by Wallman and Sandberg, (2018). The extension of the AV node model was guided by a distribution-based sensitivity analysis (Pianosi and Wagener 2018) and incorporates ANS-induced changes in the computation of the refractoriness and conduction delay. The extended model is evaluated with respect to its ability to replicate changes in heart rate and RR series variability and irregularity observed during head-up and head-down tilt test.

## 2 Materials and methods

First, the clinical tilt test data is described in Section 2.1. The RR series characteristics are defined in Section 2.2, followed by the description of a network model of the AV node (Section 2.3). A sensitivity analysis on the AV node model is described in Section 2.4, that identifies the influence of changes in model parameters on the RR series characteristics. Based on the sensitivity analysis, the AV node model is modified to account for ANS-induced changes in AV node characteristics (Section 2.5). The ability of the modified AV node model to replicate ANS induced changes in RR series characteristics observed during tilt-test is assessed in Section 2.6. Finally, the statistical analysis is described in Section 2.7, that is used to determine significant differences in AFR and RR series characteristics between tilt positions.

### 2.1 Tilt test study

The autonomic influence on the RR series characteristics was analysed using ECG data recorded during a tilt test study performed by Östenson et al. (2017). Recordings from 24 patients with persistent AF were considered of sufficient quality for analysis and were included in the present study; their age was 66 ± 9 (mean ± std), and 63% were men. None of the patients had abnormal levels of thyroid hormones, severe renal failure requiring dialysis, or heart valve disease. None of the patients were ablated for AF or on any of the Class I or Class III antiarrhythmic drugs. The tilt test was performed between 1 and 3 p.m. in a quiet study room. Standard 12-lead ECG was recorded during supine position, followed by head-down tilt (HDT, -30°) and then head-up tilt (HUT, +60°). The tilt table was manually operated and had hand grip and ankle support for HDT and foot board support for HUT; the patients remained in each position approximately 5 min. ECG preprocessing and R-peak detection was performed using the CardioLund ECG parser (www.cardiolund.com).

### 2.2 RR series characteristics

The RR series consists of the intervals between consecutive heartbeats, where the time of a heartbeat is determined by the corresponding R peak in the ECG signal. In this work, three statistical measures of the RR series characteristics were used, quantifying heart rate, heart rate variability and heart rate irregularity, respectively, defined according to Eqs. 1–3. The mean of the RR intervals $\left(\bar{RR}\right)$ is computed as

$$\bar{RR}=\frac{1}{N}\overset{N}{\underset{i=1}{\sum }}RR_{i},$$ (1)

where *RR*_*i*_ denotes the *i:*th RR interval in the RR series. The root mean square of successive RR interval differences (*RR*_*V*_, variability) is computed as

$$RR_{V}=\sqrt{\frac{1}{N-1}\overset{N-1}{\underset{i=1}{\sum }}{\left(RR_{i+1}-RR_{i}\right)}^{2}}.$$ (2)

The sample entropy of the RR series (*RR*_*I*_, irregularity) is computed as

$$RR_{I}=-\ln\left(\frac{\sum_{i=1}^{N-m}\sum_{j=1,j\neq i}^{N-m}b_{i,j}^{m+1}\left(r\right)}{\sum_{i=1}^{N-m}\sum_{j=1,j\neq i}^{N-m}b_{i,j}^{m}\left(r\right)}\right),$$ (3)

where the binary variable $b_{i,j}^{l}\left(r\right)$ with $l\in \left\{m\right.$ , $\left.m+1\right\}$ has the value 1 if the maximum absolute distance between corresponding scalar elements in the vectors $V_{i}^{l}=\left\{RR_{i}\right.$ , *RR*_*i*+1_, …, $\left.RR_{i+l-1}\right\}$ and $V_{j}^{l}$ is below the tolerance *r* times the standard deviation of the RR interval series, otherwise the value is zero (Richman and Moorman, 2000). In this study, the parameters were set to *m* = 2 and *r* = 0.2.

### 2.3 Network model of the human atrioventricular node

**FIGURE 1:**
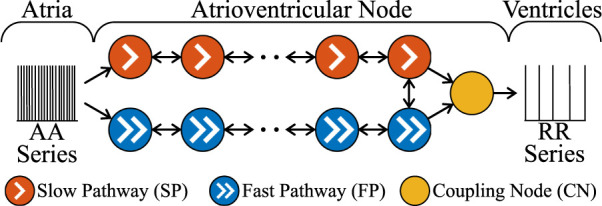
A schematic representation of the AV node model. Note that retrograde conduction is possible within the AV node model. For simplicity, only a subset of the ten nodes in each pathway is shown.

The AV node is modelled by a network of 21 nodes (cf. Figure 1) (Wallman and Sandberg, 2018; Karlsson et al., 2021). The AV nodal dual-pathway physiology with a slow pathway (SP) and a fast pathway (FP) is represented with two chains of 10 nodes each. The last nodes of the two pathways are connected to each other and to an additional coupling node (CN). Impulses enter the AV node model simultaneously at the first node of each pathway and leave the model over the CN. Retrograde conduction is possible due to the bidirectional conduction within the pathways and between the last nodes of SP and FP.

Each node represents a section of the AV node and is described with an individual refractory period *R* ^*P*^(Δ*t*_*k*_) and conduction delay *D* ^*P*^(Δ*t*_*k*_) defined as

$$R^{P}\;\left(\Delta t_{k}\right)=R_{\min}^{P}+\Delta R^{P}\left(1-e^{-\Delta t_{k}/\tau_{R}^{P}}\right),$$ (4)

$$D^{P}\;\left(\Delta t_{k}\right)=D_{\min}^{P}+\Delta D^{P}e^{-\Delta t_{k}/\tau_{D}^{P}},$$ (5)

where P ∈ {*SP*, *FP*, *CN*} denotes the association to a pathway. The electrical excitation propagation through the AV node is modelled as a series of impulses that can either be passed on or blocked by a node. This decision is based on the interval Δ*t*_*k*_ between the *k*:th impulse arrival time *t*_*k*_ and the end of the (*k*–1):th refractory period computed as

$$\Delta t_{k}=t_{k}-t_{k-1}-R^{P}\;\left(\Delta t_{k-1}\right).$$ (6)

If Δ*t*_*k*_ is positive, the impulse is conducted to all adjacent nodes, otherwise the impulse is blocked due to the ongoing refractory period *R* ^*P*^(Δ*t*_*k*−1_). The conduction delay *D* ^*P*^(Δ*t*_*k*_) describes the time delay between the arrival of an impulse at a node and its transmission to all adjacent nodes. If an impulse is conducted, *R* ^*P*^(Δ*t*_*k*_) and *D* ^*P*^(Δ*t*_*k*_) of the current node are updated according to Eqs. 4–6. For the computation of *R* ^*P*^(Δ*t*_*k*_) and *D* ^*P*^(Δ*t*_*k*_), the nodes in each pathway are characterized by six parameters, defining minimum refractory period, $R_{\min}^{P}$ ; maximum prolongation of refractory period, Δ*R* ^*P*^; time constant $\tau_{R}^{P}$ ; minimum conduction delay, $D_{\min}^{P}$ ; maximum prolongation of conduction delay, Δ*D* ^*P*^; and the time constant $\tau_{D}^{P}$ . The SP, FP and CN are modelled with separate vectors $\theta^{P}=\left[\;\right.R_{\min}^{P}$ , Δ*R* ^*P*^, $\tau_{R}^{P}$ , $D_{\min}^{P}$ , Δ*D* ^*P*^, $\tau_{D}^{P}$ ], all with fixed values.

The AV node model processes the impulse propagation chronologically and node by node, using a priority queue of nodes, sorted by impulse arrival time; details can be found in Wallman and Sandberg (2018). The input to the AV node model is a series of atrial impulses that is used to initialize the priority queue. As the impulses are conducted to adjacent nodes, new entries are added to the priority queue. The output of the AV node model is a series of impulses activating the ventricles.

In this study, the series of atrial impulses during AF is modelled as a point-process with independent inter-arrival times according to a Pearson Type IV distribution (Climent et al., 2011a). Hence, the atrial activation (AA) series is completely characterized by four parameters, namely the mean *μ*, standard deviation *σ*, skewness *γ* and kurtosis *κ*.

### 2.4 Distribution-based sensitivity analysis

The sensitivity of the three RR series characteristics $y={\left[\bar{RR},RR_{V},RR_{I}\right]}^{T}$ to the AV node model and AA series parameters $x={\left[\theta^{SP},\theta^{FP},\mu ,\sigma \right]}^{T}$ is evaluated by applying a distribution-based sensitivity analysis, based on the work of Pianosi and Wagener, (2018). For the sensitivity analysis, cumulative distribution functions (CDF) are estimated using a dataset of *K* = 250 000 randomly generated model parameter sets ***x*** and the characteristics ***y*** of the corresponding simulated RR series. For each simulation, an atrial impulse series with 60 000 AA intervals was generated using the Pearson Type IV distribution, with *μ* randomly drawn from U[100,250] ms, *σ* randomly drawn from U[15,30] ms, and *γ* and *κ* kept fixed to 1 and 6, respectively. The *γ* and *κ* were kept fixed since they cannot be estimated from the f-waves of the ECG. Negative AA intervals were excluded from the impulse series. The model parameters ***θ*** ^*SP*^ and ***θ*** ^*FP*^ were randomly drawn from bounded uniform distributions given in Table 1, as previously done in Karlsson et al. (2021). The ***θ*** ^*CN*^ were kept fixed according to Table 1, corresponding to *R* ^*P*^(Δ*t*_*k*_) and *D* ^*P*^(Δ*t*_*k*_) of the CN equal to 250 ms and 0 ms, respectively.

**TABLE 1:** Model parameters used for the sensitivity analysis.

Parameters	SP (ms)	FP (ms)	CN (ms)
*R*_min_	U[250,600]	U[250,600]	250
Δ*R*	U[0,600]	U[0,600]	0
*τ*_*R*_	U[50,300]	U[50,300]	1
*D*_min_	U[0,30]	U[0,30]	0
Δ*D*	U[0,75]	U[0,75]	0
*τ*_*D*_	U[50,300]	U[50,300]	1

The RR series characteristics were computed using a series of 4000 RR intervals corresponding to the first impulses that left the AV node model through the CN. Two selection criteria were used to remove non-physiological parameter sets. First, a model parameter set was only included if the slow pathway had a lower refractory period *R* ^*SP*^(Δ*t*_*k*_) < *R* ^*FP*^(Δ*t*_*k*_) and higher conduction delay *D* ^*SP*^(Δ*t*_*k*_) > *D* ^*FP*^(Δ*t*_*k*_) than the fast pathway for all Δ*t*_*k*_. Second, the resulting $\bar{RR}$ was required to be in the range 300  ms $\leq \bar{RR}\leq$ 1000 ms, corresponding to heart rates between 60 bpm and 200 bpm. Heart rates below 60 bpm are disregarded, because the pacemaker function of the AV node, that becomes relevant in this case (George et al., 2017), is not incorporated in the AV node model. Heart rates above 200 bpm are disregarded based on a reported minimum refractory period in the bundle branches of around 300 ms (Denes et al., 1974).

**FIGURE 2:**
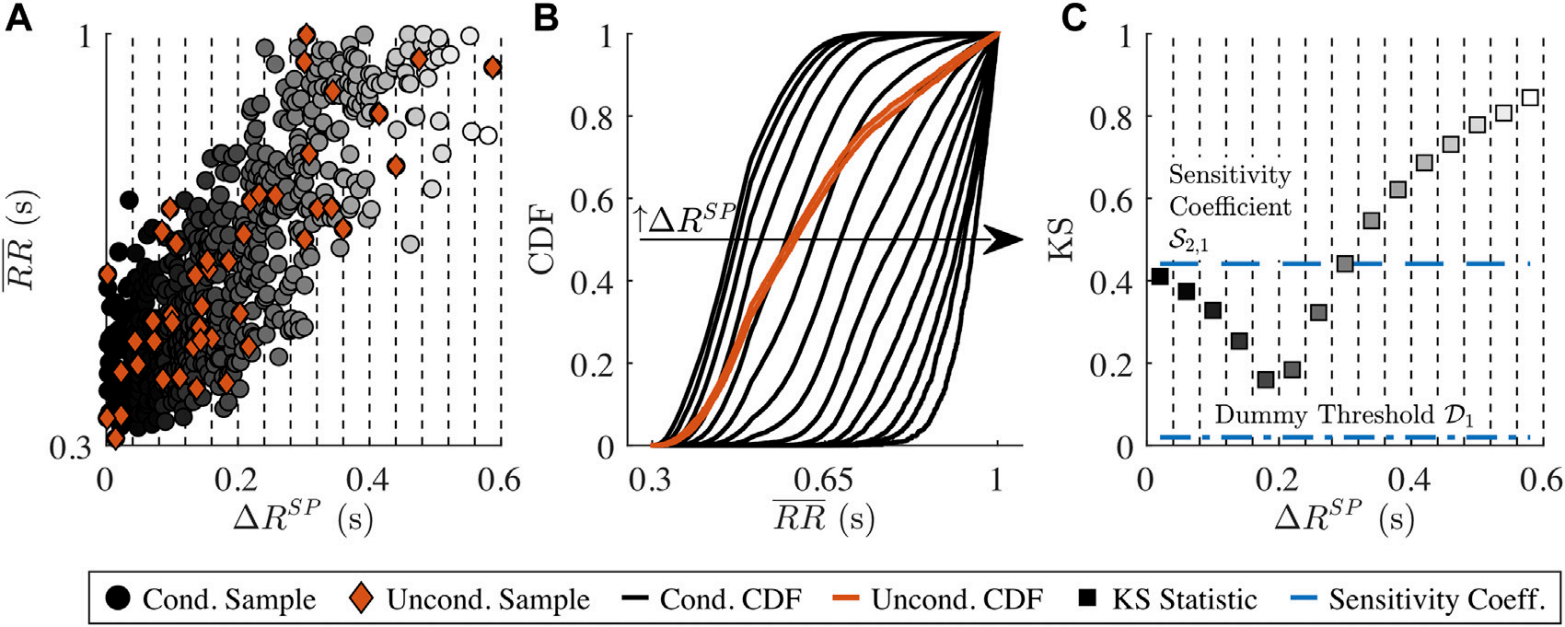
Illustration of the distribution-based sensitivity analysis. **(A)** $\bar{RR}$ plotted against one of the model parameters Δ*R* ^*SP*^. The samples that are used to estimate the conditional CDFs $F_{y_{m}|x_{n}}\left(y_{m}|x_{n}\in I_{c}\right)$ are illustrated as circles and the conditioning intervals $I_{c}$ are illustrated with vertical dotted lines. The samples that are used to estimate the unconditional CDF $F_{y_{m}}^{\left(d\right)}\left(y_{m}\right)$ are illustrated as diamonds. **(B)** $F_{y_{m}|x_{n}}\left(y_{m}|x_{n}\in I_{c}\right)$ are illustrated as black lines, where the leftmost line corresponds to $F_{y_{m}|x_{n}}\left(y_{m}|x_{n}\in I_{1}\right)$ with the lowest Δ*R* ^*SP*^ values and the rightmost line corresponds to $F_{y_{m}|x_{n}}\left(y_{m}|x_{n}\in I_{15}\right)$ . The 1,000 $F_{y_{m}}^{\left(d\right)}\left(y_{m}\right)$ lay all within the area illustrated by the red patch. **(C)** Each of the 15 squares correspond to $\underset{d=1,\ldots ,D}{median}\;\;KS\left(F_{y_{m}}^{\left(d\right)}\left(y_{m}\right),F_{y_{m}|x_{n}}\left(y_{m}|x_{n}\in I_{c}\right)\right)$ .

A sensitivity coefficient $S_{n,m}$ is computed for each pair of model parameter *x*_*n*_ and RR series characteristic *y*_*m*_, where *x*_*n*_ is the *n*:th element in ***x*** and *y*_*m*_ is the *m*-th element in ***y***. The $S_{n,m}$ indicates how much a change in model parameter *x*_*n*_ affects the distribution of *y*_*m*_ and is defined as

$$S_{n,m}=\underset{c=1,\ldots ,C}{median}\;\;\underset{d=1,\ldots ,D}{median}\;\;KS\left(F_{y_{m}}^{\left(d\right)}\left(y_{m}\right),F_{y_{m}|x_{n}}\left(y_{m}|x_{n}\in I_{c}\right)\right),$$ (7)

where $KS\left(F_{y_{m}}^{\left(d\right)}\left(y_{m}\right),F_{y_{m}|x_{n}}\left(y_{m}|x_{n}\in I_{c}\right)\right)$ is the Kolmogorov-Smirnov (KS) distance between the unconditional CDF $F_{y_{m}}^{\left(d\right)}\left(y_{m}\right)$ and the conditional CDF $F_{y_{m}|x_{n}}\left(y_{m}|x_{n}\in I_{c}\right)$ . When estimating $F_{y_{m}|x_{n}}\left(y_{m}|x_{n}\in I_{c}\right)$ , the range of variation of *x*_*n*_ is split into *C* = 15 equally spaced conditioning intervals $I_{c}$ , with *c* = 1, …, *C* (cf. Figure 2A). All samples within $I_{c}$ are used to estimate the corresponding $F_{y_{m}|x_{n}}\left(y_{m}|x_{n}\in I_{c}\right)$ (cf. Figure 2B). To generate the set of $F_{y_{m}}^{\left(d\right)}\left(y_{m}\right)$ , with *d* = 1, …, *D*, a subset of *K*/*C* samples are bootstrapped *D* = 1000 times (cf. Figures 2A,B). The KS distance is defined as

$$KS\left(F_{1}\left(y\right),F_{2}\left(y\right)\right)=\underset{y}{max}\left|F_{1}\left(y\right)-F_{2}\left(y\right)\right|.$$ (8)

As the $F_{y_{m}}^{\left(d\right)}\left(y_{m}\right)$ and $F_{y_{m}|x_{n}}\left(y_{m}|x_{n}\in I_{c}\right)$ are approximations based on a finite number of samples, parameters that have no influence on *y*_*m*_ can result in $S_{n,m}$ above zero. The impact of approximation errors on $S_{n,m}$ can be estimated for each *y*_*m*_ using a dummy parameter $D_{m}$ defined as

$$D_{m}=\underset{d=2,\ldots ,D}{median}\;\;KS\left(F_{y_{m}}^{\left(d\right)}\left(y_{m}\right),F_{y_{m}}^{\left(1\right)}\left(y_{m}\right)\right),$$ (9)

A model parameter *x*_*n*_ is determined to have influence on *y*_*m*_ if and only if $S_{n,m}>D_{m}$ .

### 2.5 Extended atrioventricular node model accounting for autonomic nervous system induced changes

The results from the sensitivity analysis (Section 3.1) indicate that changes in both the AV node model parameters and the AA series parameters have an influence on the RR series characteristics. Based on this, the AV node model described in Section 2.3 is extended to account for ANS-induced changes in the AA series by allowing *μ*(*t*) and *σ*(*t*) of the Pearson Type IV distribution to vary over time. Moreover, the AV node model was extended by two scaling factors *A*_*R*_ and *A*_*D*_, accounting for the effect of changes in autonomic tone on refractory period (*A*_*R*_) and on conduction delay (*A*_*D*_).

$$R^{P}\;\left(\Delta t_{k},A_{R}\right)=A_{R}\cdot R^{P}\;\left(\Delta t_{k}\right)=A_{R}\left(R_{\min}^{P}+\Delta R^{P}\left(1-e^{-\Delta t_{k}/\tau_{R}^{P}}\right)\right)$$ (10)

$$D^{P}\;\left(\Delta t_{k},A_{D}\right)=A_{D}\cdot D^{P}\;\left(\Delta t_{k}\right)=A_{D}\left(D_{\min}^{P}+\Delta D^{P}e^{-\Delta t_{k}/\tau_{D}^{P}}\right)$$ (11)

The factors *A*_*R*_ and *A*_*D*_ model the combined effect of changes in sympathetic and parasympathetic activity and do not differ between the SP, FP and CN.

### 2.6 Tilt-induced changes in extended atrioventricular node model

In this section, the extended AV node model proposed in Section 2.5 is investigated with respect to its ability to mimic tilt-induced changes in RR series characteristics.

**FIGURE 3:**
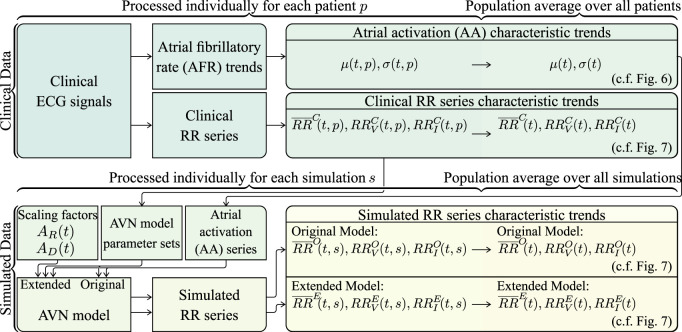
Schematic illustrating how the clinical and simulated RR series characteristic trends are computed.

**FIGURE 4:**
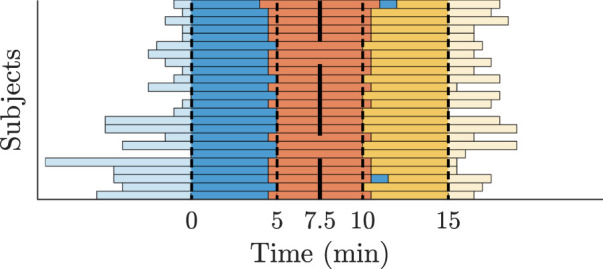
Tilt recordings of 24 patients divided into supine (blue), HDT (red) and HUT (yellow). The bars represent the length of the recorded ECG data. All recordings are centered along the middle of the HDT section.

The clinical ECG signals (cf. Section 2.1) are used to generate AA series for the AV node model input and to compare the characteristics of the simulated RR series to the clinical RR series (cf. Figure 3). For this purpose, a continuous 15-min ECG signal with 5 minutes per supine, HDT and HUT position was desired for each patient. In the clinical data, however, the length of the three tilt positions varied between patients with the supine position being between 5 and 13 min, HDT being between 5 and 7 min and HUT being between 5 and 9 min. For two patients, there was an additional minute in supine position between the HDT and HUT. The ECG signals were aligned to the middle of the HDT section and a 15-min long segment centered around the same midpoint was chosen for each patient (cf. Figure 4).

The clinical RR series characteristic trends ${\bar{RR}}^{C}\;\;\left(t,p\right)$ , $RR_{V}^{C}\left(t,p\right)$ and $RR_{I}^{C}\;\left(t,p\right)$ for each patient *p* are computed from the RR intervals using a sliding window of length *N* according to Eqs. 1–3 (cf. Figure 3). For *RR*_*V*_ and *RR*_*I*_, *N* is set to 200, because shorter RR interval series might lead to inaccuracies in the sample entropy computation (Yentes et al., 2013). For $\bar{RR}$ , *N* is set to 100, as its computation is more robust than the computation of *RR*_*V*_ and *RR*_*I*_ and shorter RR interval series allow for a better temporal resolution. RR intervals in the clinical RR series preceding and following ectopic beats were excluded. For the computation of *RR*_*I*_ according to Eq. 3, vectors $V_{i}^{l}$ with excluded RR intervals were omitted. The RR series characteristic trends of each patient ${\bar{RR}}^{C}\;\;\left(t,p\right)$ , $RR_{V}^{C}\left(t,p\right)$ and $RR_{I}^{C}\;\left(t,p\right)$ were averaged over all 24 patients to obtain population-averaged clinical trends ${\bar{RR}}^{C}\;\;\left(t\right)$ , $RR_{V}^{C}\left(t\right)$ and $RR_{I}^{C}\;\left(t\right)$ (cf. Figure 3).

For the generation of the AA series, first, an atrial fibrillatory rate (AFR) trend is estimated from each 15-min ECG segment (cf. Figure 3). The AFR is estimated by fitting a complex sinusoidal model to the f-waves of the ECG, following spatiotemporal QRST cancellation, as described in Henriksson et al. (2018). From each of the resulting AFR trends, the AA series parameters *μ*(*t*, *p*) and *σ*(*t*, *p*) are estimated by the mean and standard deviation of 1/AFR using 1-min sliding windows; the resolution of the AFR trend is 0.02 s (cf. Figure 3). Then, *μ*(*t*, *p*) and *σ*(*t*, *p*) are averaged over all 24 patients, resulting in the population-averaged trends *μ*(*t*) and *σ*(*t*) (cf. Figure 3). Finally, the AA series is iteratively generated (cf. Figure 3). The first AA interval is drawn from the Pearson Type IV distribution with *μ*(0) and *σ*(0), and each consecutive AA interval is drawn from the distribution with *μ*(*t*_*i*_) and *σ*(*t*_*i*_) where *t*_*i*_ corresponds to the accumulated time of the previous AA intervals. The *γ* and *κ* of the Pearson Type IV distribution were kept fixed to 1 and 6, respectively.

For the simulations using the original and extended model, a set of 240 AV node model parameter vectors $x^{\prime }={\left[\theta^{SP},\theta^{FP},\theta^{CN}\right]}^{T}$ were generated (cf. Figure 3). Ten parameter vectors per patient were selected from a set of randomly drawn parameter sets based on their ability to replicate the RR series characteristics of the 5-min long supine segment of the respective patient. A detailed description of the parameter sets and the selection process can be found in the Supplementary Section 1. The ranges of the model parameters in the 240 parameter sets are given in Table 2.

**TABLE 2:** Ranges of the 240 model parameters used for the illustration (mean ± std).

Parameters	SP (ms)	FP (ms)	CN (ms)
*R*_min_	339 ± 77	493 ± 82	250 ± 0
Δ*R*	232 ± 112	369 ± 161	0 ± 0
*τ*_*R*_	160 ± 77	162 ± 72	1 ± 0
*D*_min_	20 ± 7	7 ± 6	0 ± 0
Δ*D*	39 ± 20	23 ± 16	0 ± 0
*τ*_*D*_	171 ± 71	163 ± 70	1 ± 0

For the computation of simulated RR series characteristic trends using the original and the extended model, respectively, simulations were performed with each of the 240 parameter sets using 10 different realizations of the AA series generated from *μ*(*t*) and *σ*(*t*). In the original model, the scaling factors *A*_*R*_ and *A*_*D*_ are not included, which is equivalent to the extended model using *A*_*R*_ = 1 and *A*_*D*_ = 1 (cf. Figure 3). In the extended model, *A*_*R*_ and *A*_*D*_ were allowed to change between supine and HDT and between HDT and HUT, respectively, but were assumed to remain constant within each position. Hence, for the extended model, *A*_*R*_ and *A*_*D*_ were set to 1 in the supine position, and different combinations of *A*_*R*_ ∈ {0.95, 1, 1.05} and *A*_*D*_ ∈ {0.8, 1, 1.2} were used for the simulations during HDT and HUT. For each simulation *s*, the mean RR interval trends ${\bar{RR}}^{O}\;\;\left(t,s\right)$ and ${\bar{RR}}^{E}\;\;\left(t,s\right)$ were computed from the RR interval series using a sliding window of length *N* = 100 (cf. Eq. 1). Whereas the RR variability and RR irregularity trends $RR_{V}^{O}\left(t,s\right)$ and $RR_{I}^{O}\;\left(t,s\right)$ , as well as $RR_{V}^{E}\left(t,s\right)$ and $RR_{I}^{E}\;\left(t,s\right)$ were computed from the RR interval series using a sliding window of length *N* = 200 (cf. Eqs. 2 and 3). The simulated RR series characteristic trends were averaged over all parameter sets and realizations to obtain the population-averaged simulated trends ${\bar{RR}}^{O}\;\;\left(t\right)$ , $RR_{V}^{O}\left(t\right)$ and $RR_{I}^{O}\;\left(t\right)$ for the original model and ${\bar{RR}}^{E}\;\;\left(t\right)$ , $RR_{V}^{E}\left(t\right)$ and $RR_{I}^{E}\;\left(t\right)$ for the extended model (cf. Figure 3).

### 2.7 Statistical analysis

A Wilcoxon signed rank test was applied to determine if AFR, $\bar{RR}$ , *RR*_*V*_ and *RR*_*I*_ differed significantly between supine, HDT and HUT. For the analysis, the AFR and RR series characteristics were computed for each patient and tilt position using the 5-min long ECG segments (cf. Figure 4). A *p*-value <0.05 was considered significant.

## 3 Results

### 3.1 Sensitivity analysis

**FIGURE 5:**
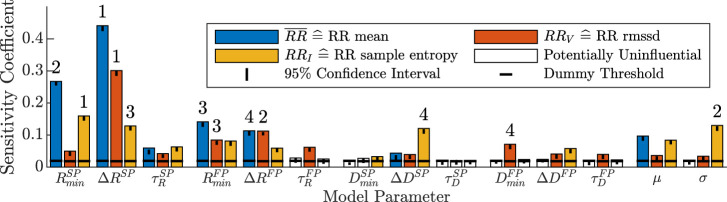
Distribution-based sensitivity indices describing the influence of changes in the 14 model parameters to changes in the three RR series characteristics. A model parameter is assumed to have influence on the RR series characteristics if the sensitivity coefficient is above the threshold of the dummy parameter (horizontal black line), otherwise it is not influential and illustrated with a white bar. The black vertical line illustrates the 95% confidence interval of the *t* bootstrapping iterations of the sensitivity coefficient. The ranking of the four most influential model parameters for each RR series characteristic is shown with the numbers above the bars.

Results from the distribution-based sensitivity analysis (described in Section 2.4) with respect to the influence of the AV node model parameters on RR series characteristics are shown in Figure 5. Heart rate, quantified by $\bar{RR}$ is predominantly sensitive to changes in the refractory period parameters with the four largest contributors being the *R*_min_ and Δ*R* parameters of both pathways. In contrast, the changes in the conduction delay had little influence on the $\bar{RR}$ , with Δ*D* ^*SP*^ being the only conduction delay parameter that is slightly above the dummy threshold. Changes in the mean of the AA series *μ* were also influential on the $\bar{RR}$ , while changes in the standard deviation *σ* of the AA series are not considered to have influence to changes in $\bar{RR}$ .

For *RR*_*V*_ quantifying RR series variability, nearly all model parameters of the refractory period, conduction delay and AA series had sensitivity coefficients above the dummy threshold. The four largest contributors to changes in the *RR*_*V*_ were the Δ*R* parameters of both pathways, as well as the minimum refractory period and minimum conduction delay of the fast pathway, $R_{\min}^{FP}$ and $D_{\min}^{FP}$ .

The *RR*_*I*_ quantifying RR series irregularity was also influenced by most model parameters of the refractory period, conduction delay and AA series. The four largest contributors were the minimum refractory period of the slow pathway $R_{\min}^{SP}$ , the standard deviation *σ* of the AA series and the maximum prolongation of the refractory period and conduction delay of the slow pathway, Δ*R* ^*SP*^ and Δ*D* ^*SP*^.

### 3.2 Clinical data

The AFR decreased significantly from the supine position to HDT and increased significantly from HDT to HUT, where the AFR during HUT was significantly higher than during supine (Table 3). The heart rate increased during HDT and increased further during HUT (Table 3). The results align with the observations of Östenson et al. (2017). The variability and irregularity of the RR series decreased during HDT and decreased further during HUT (Table 3). For the variability and irregularity of the RR series, only the differences between supine and HUT were statistically significant.

**TABLE 3:** Mean ± std of AFR and RR series characteristics of the 24 patients in the study population for each tilt position.

Tilt Position	Supine	HDT	HUT
AFR mean (Hz)	6.78 ± 0.64	6.62 ± 0.7^**^	6.84 ± 0.63^*,†^
$\bar{RR}\;\left(ms\right)$	656 ± 126	642 ± 111^*^	613 ± 115^**,†^
*RR*_*V*_ (ms)	192 ± 54	182 ± 45	176 ± 51^**^
*RR*_*I*_	2.09 ± 0.2	2.05 ± 0.28	1.95 ± 0.31^**^

HDT, head-down tilt; HUT, head-up tilt. ^*^ *p* < 0.05 vs Supine. ^**^ *p* < 0.01 vs Supine. ^†^ *p* < 0.05 vs HDT.

### 3.3 Tilt-induced changes in atrioventricular node model

**FIGURE 6:**
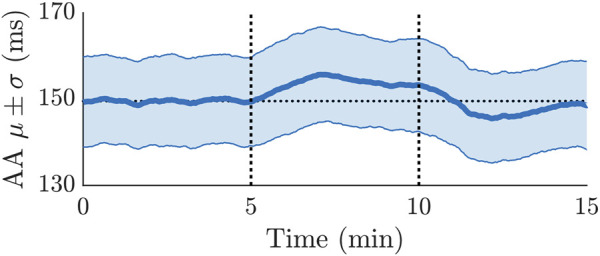
Averaged mean and standard deviation of the AA series estimated from ECG recordings of 24 patients. The first 5 min were during supine position, followed by 5 min of HDT and 5 min of HUT. The horizontal dotted line illustrates the average of the *μ* trend during the first 5 min.

The average of *μ*(*t*) and *σ*(*t*) over all 24 patients is illustrated in Figure 6. The *μ*(*t*) shows a clear variation during HDT and HUT, but not in supine position, where *μ*(*t*) was approximately constant around 150 ms. Compared to *μ*(*t*) during supine position, *μ*(*t*) increased during HDT and decreased during HUT (cf. Figure 6).

**FIGURE 7:**
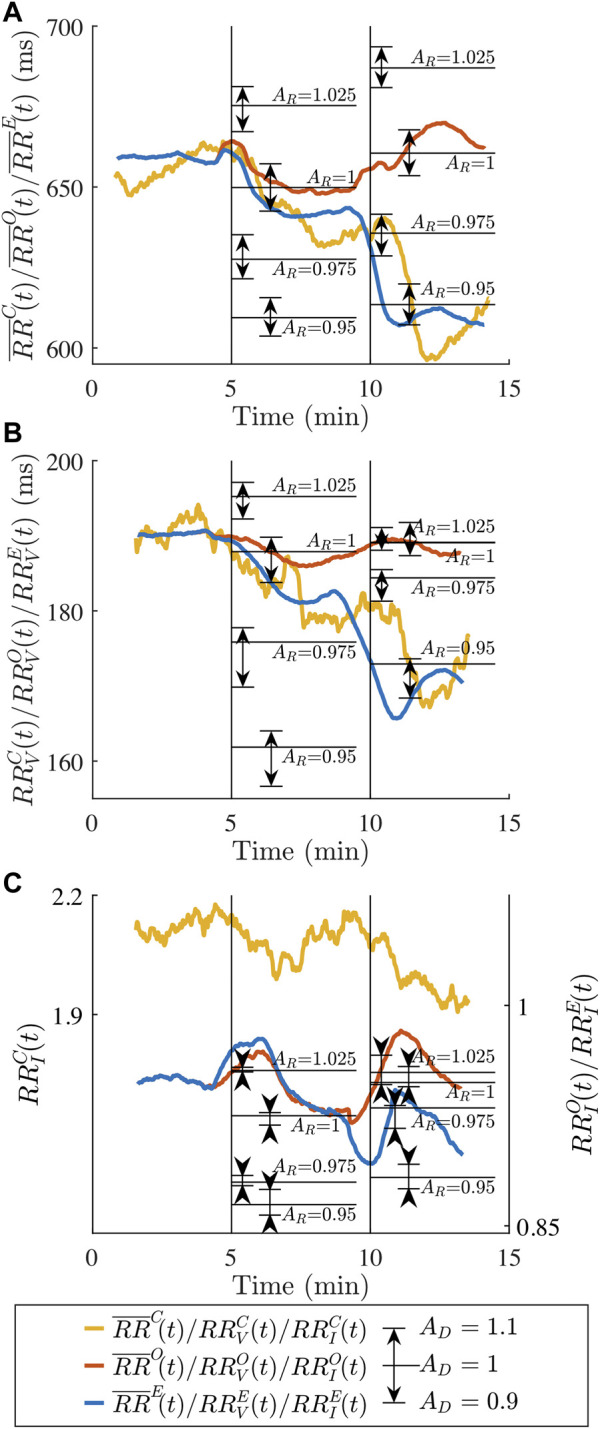
Average clinical RR series characteristics **(A)** ${\bar{RR}}^{C}\;\;\left(t\right)$ **(B)** $RR_{V}^{C}\left(t\right)$ and **(C)** $RR_{I}^{C}\;\left(t\right)$ (yellow) and average simulated RR series characteristics for the original model **(A)** ${\bar{RR}}^{O}\;\;\left(t\right)$ **(B)** $RR_{V}^{O}\left(t\right)$ and **(C)** $RR_{I}^{O}\;\left(t\right)$ (red) and average simulated RR series characteristics for the extended model **(A)** ${\bar{RR}}^{E}\;\;\left(t\right)$ **(B)** $RR_{V}^{E}\left(t\right)$ and **(C)** $RR_{I}^{E}\;\left(t\right)$ (blue). The dashed black lines mark the transition between the supine and HDT, and HDT and HUT, respectively. Horizontal black lines show 5-min averages of ${\bar{RR}}^{E}\;\;\left(t\right)$ , $RR_{V}^{E}\left(t\right)$ and $RR_{I}^{E}\;\left(t\right)$ during HDT or HUT with *A*_*R*_ as indicated and *A*_*D*_ = 1. Arrows show the impact of perturbing *A*_*D*_ by +0.1 (arrow pointing up) or −0.1 (arrow pointing down).

In Figure 7, the characteristics ${\bar{RR}}^{C}\;\;\left(t\right)$ , $RR_{V}^{C}\left(t\right)$ and $RR_{I}^{C}\;\left(t\right)$ estimated from clinical data during tilt test are illustrated. It can be seen that ${\bar{RR}}^{C}\;\;\left(t\right)$ , $RR_{V}^{C}\left(t\right)$ and $RR_{I}^{C}\;\left(t\right)$ are decreasing from supine to HDT and decreasing further from HDT to HUT. When performing the simulations with the original model, ${\bar{RR}}^{O}\;\;\left(t\right)$ , $RR_{V}^{O}\left(t\right)$ and $RR_{I}^{O}\;\left(t\right)$ are decreasing from supine to HDT, but increasing from HDT to HUT. When performing the simulations with the extended model, ${\bar{RR}}^{E}\;\;\left(t\right)$ , $RR_{V}^{E}\left(t\right)$ and $RR_{I}^{E}\;\left(t\right)$ are decreasing from supine to HDT and decreasing further from HDT to HUT. Comparing the clinical and simulated trends of $\bar{RR}\left(t\right)$ and *RR*_*V*_(*t*), it can be seen that the extended model accounting for ANS-induced changes can better replicate the observed changes to the clinical RR series characteristics compared to the original model. For *RR*_*I*_(*t*), both the original and extended model produce RR series that are more regular than the clinical RR series, as the irregularity quantified by the sample entropy is higher for the clinical RR series. For the simulated RR series characteristics of the extended model, the average of ${\bar{RR}}^{E}\;\;\left(t\right)$ , $RR_{V}^{E}\left(t\right)$ and $RR_{I}^{E}\;\left(t\right)$ during the 5 min in HDT and HUT were illustrated for the nine different combinations of *A*_*R*_ ∈ {0.95, 1, 1.05} and *A*_*D*_ ∈ {0.9, 1, 1.1}. For ${\bar{RR}}^{E}\;\;\left(t\right)$ , $RR_{V}^{E}\left(t\right)$ and $RR_{I}^{E}\;\left(t\right)$ , an increase in *A*_*R*_ causes an increase, and for ${\bar{RR}}^{E}\;\;\left(t\right)$ and $RR_{V}^{E}\left(t\right)$ , an increase in *A*_*D*_ causes an increase. However, for $RR_{I}^{E}\;\left(t\right)$ , an increase in *A*_*D*_ instead causes a decrease. The ${\bar{RR}}^{E}\;\;\left(t\right)$ , $RR_{V}^{E}\left(t\right)$ and $RR_{I}^{E}\;\left(t\right)$ are obtained using *A*_*R*_ = 1 and *A*_*D*_ = 0.9 during HDT and *A*_*R*_ = 0.95 and *A*_*D*_ = 0.9 during HUT and are displayed in Figure 7; the scaling factors were chosen so that the resulting ${\bar{RR}}^{E}\;\;\left(t\right)$ and $RR_{V}^{E}\left(t\right)$ matches ${\bar{RR}}^{C}\;\;\left(t\right)$ and $RR_{V}^{C}\left(t\right)$ .

## 4 Discussion

The aim of this study was to extend the AV node model (Wallman and Sandberg, 2018) to incorporate ANS-induced changes. The extension of the AV node model was guided by a distribution-based sensitivity analysis. The sensitivity analysis indicated that the refractory period and conduction delay parameters as well as the atrial impulse series had a significant influence on the heart rate as well as the variability and the irregularity of the RR series, while the most influential parameters were predominantly those describing the refractory period. Rather than modelling the effect of the sympathetic and parasympathetic activity separately, we describe the joint effects, i.e., the autonomic tone. We proposed an extension to the AV node model that accounts for the ANS-induced changes by introducing scaling factors for the refractory period and conduction delay. The capability of the extended AV node model to replicate ANS-induced changes was investigated by comparison to ECG data acquired during tilt test.

Our results (Figure 7) indicate that the extended model, but not the original, could replicate the observed changes in the clinical RR series characteristics during HUT and HDT, since the changes in RR series characteristics could not be explained by changes in atrial activity alone. The ${\bar{RR}}^{E}\;\;\left(t\right)$ , $RR_{V}^{E}\left(t\right)$ and $RR_{I}^{E}\;\left(t\right)$ (Figure 7) show that a decrease in refractory period and conduction delay allow the model to replicate the decrease in ${\bar{RR}}^{C}\;\;\left(t\right)$ , $RR_{V}^{C}\left(t\right)$ and $RR_{I}^{C}\;\left(t\right)$ . Conversely, if the refractory period and conduction delay are kept fixed for ${\bar{RR}}^{O}\;\;\left(t\right)$ , $RR_{V}^{O}\left(t\right)$ and $RR_{I}^{O}\;\left(t\right)$ , all three RR series characteristics increase during HUT, which is the opposite direction of change of ${\bar{RR}}^{C}\;\;\left(t\right)$ , $RR_{V}^{C}\left(t\right)$ and $RR_{I}^{C}\;\left(t\right)$ . When comparing $RR_{I}^{O}\;\left(t\right)$ and $RR_{I}^{E}\;\left(t\right)$ with $RR_{I}^{C}\;\left(t\right)$ , it can be seen that the sample entropy of the simulated RR series is lower than that of the clinical RR series. This highlights that the simulated RR series are more regular than the clinical RR series. One possible explanation for a lower irregularity in simulated RR series is the lack of short-term variations in AV node refractoriness and conduction delay. Such short-term variations may be induced by respiratory modulation in ANS activity. Thus, a natural next step in our model development will be to incorporate the respiratory modulation of the ANS, likely via periodical variations in the scaling factors *A*_*R*_ and *A*_*D*_.

Many electrophysiological (EP) studies have demonstrated that an increase in sympathetic activity is causing a decrease in the human AV nodal conduction delay (Lister et al., 1965; Dhingra et al., 1973; Morady et al., 1988; Cossú et al., 1997) and a decrease in the refractory period (Morady et al., 1988; Cossú et al., 1997). Moreover, a decrease in sympathetic activity in the human AV node is causing an increase in conduction delay and refractory period (Morady et al., 1988). Head-up tilt is associated with increased sympathetic tone, and it has been demonstrated that the AV nodal conduction delay and refractory period decrease when changing the posture from supine to standing (Hashimoto et al., 1991). The results in Figure 7 confirm that a reduction in the conduction delay using *A*_*D*_ = 0.9 and a reduction in the refractory period using *A*_*R*_ = 0.95 better replicate the observed changes in the clinical RR series characteristics than the original model during HUT. Decreases in refractory period and conduction delay of up to 30% in response to isoproterenol-induced increases in sympathetic activity have been reported (Lister et al., 1965; Dhingra et al., 1973; Cossú et al., 1997). However, when considering that the reported changes in heart rate due to the isoproterenol administration is larger than the observed changes in $\bar{RR}$ during tilt, the parameter choice of *A*_*D*_ = 0.9 and *A*_*R*_ = 0.95 are reasonable for the tilt test data used in this study.

Increased parasympathetic activity has been associated with an increased conduction delay (Martin, 1977); studies in dogs reported an increased conduction delay with acetylcholine administration (Priola et al., 1983; Bertrix et al., 1984) and vagal stimulation (Spear and Moore, 1973; Martin, 1975; Pirola and Potter, 1990). Moreover, there are indications that an increased parasympathetic activity is associated with an increased refractory period (Martin, 1977); experimental studies using rabbit hearts reported an increased AV-nodal refractory period (West and Toda, 1967) and occurrences of 2:1 AV nodal block (Cranefield et al., 1959) with acetylcholine administration, and studies in dogs reported occurrences of AV block with acetylcholine administration (Hageman et al., 1985) and vagal stimulation (Spear and Moore, 1973; Hageman et al., 1985).

It is unclear how the HDT affects the sympathetic and parasympathetic activity. The results in Figure 7 show that a reduction in the conduction delay using *A*_*D*_ = 0.9 and no modification of the refractory period using *A*_*R*_ = 1 better replicate the observed changes in the clinical RR series characteristics than the original model during HDT. These results are consistent with possible slight increase in sympathetic tone provoked by HDT. However, other interpretations are possible. Nagaya et al. (1995) postulated a diminished sympathetic activity in HDT. Under that hypothesis, the results in Figure 7 suggest a decrease in parasympathetic tone to revert the direction of change caused by a decreased sympathetic tone. It should be noted that the model presented here does not distinguish between these two possibilities, since *A*_*D*_ and *A*_*R*_ are modelling the joint effect of changes in parasympathetic and sympathetic activity. Hence, the scale factor *A*_*D*_ = 0.9 during HDT could be reflecting either a slight increase in sympathetic activity, a slight decrease in parasympathetic activity, a larger increase in sympathetic activity combined with an increase in parasympathetic activity, or a large decrease in parasympathetic activity combined with a decrease in sympathetic activity.

The set of scaling factors *A*_*R*_ and *A*_*D*_ used to create ${\bar{RR}}^{E}\;\;\left(t\right)$ , $RR_{V}^{E}\left(t\right)$ and $RR_{I}^{E}\;\left(t\right)$ in Figure 7 results in RR series characteristics similar to that observed during HDT and HUT. The results in Figure 7 show that a scaling factor *A*_*R*_ below 1, i.e., a decrease of the refractory period, causes a decrease in ${\bar{RR}}^{E}\;\;\left(t\right)$ , $RR_{V}^{E}\left(t\right)$ and $RR_{I}^{E}\;\left(t\right)$ . Conversely, a scaling factor *A*_*R*_ above 1, i.e., an increase of the refractory period, causes an increase in ${\bar{RR}}^{E}\;\;\left(t\right)$ , $RR_{V}^{E}\left(t\right)$ and $RR_{I}^{E}\;\left(t\right)$ . A scaling factor *A*_*D*_ below 1, i.e., a decrease in conduction delay, causes a decrease in ${\bar{RR}}^{E}\;\;\left(t\right)$ , $RR_{V}^{E}\left(t\right)$ and vice versa. The opposite relationship can be seen for the RR series irregularity, where a scaling factor *A*_*D*_ below 1 causes an increase in $RR_{I}^{E}\;\left(t\right)$ and vice versa. Moreover, when considering one RR series characteristic at a time, it can be anticipated in Figure 7 that the same 5-min average value of the RR series characteristics can be achieved with different combinations of *A*_*R*_ and *A*_*D*_. Hence, considering all three RR series characteristics simultaneously increases the likelihood of identifying a unique pair of scaling factors *A*_*R*_ and *A*_*D*_ that fits the observed data.

To reduce the complexity of the model, the refractoriness and conduction delay of the SP, FP and CN are modified with the same *A*_*R*_ and *A*_*D*_. However, due to the structural and molecular heterogeneity of the different pathways, it is likely that the ANS-induced changes affect each pathway differently (George et al., 2017). In rabbit hearts, it was reported that acetylcholine strongly affects fibers of the atrionodal junction but does not show any effect in the lower part of the node or the bundle of His (Trautwein, 1963). In the description of the AV node model, the CN is merging the impulses from the SP and FP and its refractory period and conduction delay is independent of Δ*t*_*k*_. In contrast to Karlsson et al. (2021), $R_{\min}^{CN}$ was set to the minimum of the bounded uniform distributions for the $R_{\min}^{SP}$ and the $R_{\min}^{FP}$ given in Table 1. Further, the conduction delay of the CN was set to 0, as other choices of a constant conduction delay would not have changed the resulting RR series. In previous work on the network model (Wallman and Sandberg, 2018; Karlsson et al., 2021), the AA interval series was modelled as a Poisson process. However, based on results of Climent et al. (2011a), a Pearson Type IV distribution better reproduces the statistical properties of the AA interval series during AF and was therefore chosen in the present study. The mean and standard deviation of the Pearson Type IV distribution were determined from the mean and standard deviation of the AFR. However, the skewness and kurtosis were fixed, as their sensitivity coefficients were uninfluential (data not shown) and since there is no straight-forward way to estimate these parameters from the f-waves of the ECG.

In the present study, the ability of the extended model to mimic tilt-induced changes was investigated using data from a previous study (Östenson et al., 2017), with tilt angles fixed to -30° in HDT and 60° in HUT, respectively. Different tilt angles of the tilt, i.e., different magnitude of the orthostatic stimulus, may affect the ANS response and hence the resulting RR series characteristics. Previous results from patients in normal sinus rhythm show that the sample entropy of the RR series was decreasing during HUT from 0° to 60° but remained roughly constant from 60° to 90° (Porta et al., 2007). Based on these results, we assume that the tilt angle of 60° is sufficiently large to induce changes in autonomic tone. Access to data from patients with AF during other tilt-inclinations could potentially be used to refine the model to take the degree on inclination into account. The tilt-induced changes in RR series irregularity observed in the present study are in line with the results in Patel et al. (2018), where a decrease in RR sample entropy in response to HUT in patients with AF was reported. The tilt-induced changes in RR series irregularity observed in the present study are also in line with the changes reported for patients in normal sinus rhythm during HUT (Porta et al., 2007). Results from previous studies suggest that the RR series irregularity during normal sinus rhythm increase in response to HDT (Porta et al., 2015), whereas a slight but not significant decrease was observed in the present study with patients in AF. However, it should be noted that origin of RR series variability and irregularity during AF differs from that during normal sinus rhythm and hence, the interpretation of the results with respect to autonomic tone may be different.

The effect of the ANS-induced activity was investigated with respect to its ability to mimic the population-averaged changes observed during tilt test. The RMSSD and sample entropy were used to quantify RR series variability and irregularity, respectively, since these statistical measures have been used in previous studies to assess changes in RR series characteristics during AF in response to drugs (Corino et al., 2015) and tilt-test (Patel et al., 2018). Population-averaged trends were chosen over the trends of individual patients to reduce the uncertainty in the estimation of the clinical *RR*_*V*_ and *RR*_*I*_ trends. The parameter sets used for the simulations in Section 2.6 were selected to be representative of the patients in the present study based on their ability to replicate RR series characteristics observed during supine position. However, it should be noted that fitting of the model to individual patients is outside the scope of the present study. Due to the short measurement duration of the clinical data, a robust estimation of individual model parameters is not to be expected with the present methodology (Karlsson et al., 2021). Longer measurements from more patients will allow model development and evaluation on a patient-specific basis, forming an attractive next step.

A distribution-based sensitivity analysis was chosen over a variance-based method, because the distributions of the simulated RR series characteristics are highly-skewed and multi-modal. Hence, variance alone cannot adequately represent the uncertainty (Pianosi and Wagener, 2018). Instead, a distribution-based method characterizes the uncertainty and sensitivity by investigating the entire distribution of the model outputs (Pianosi and Wagener, 2018). The results of the sensitivity analysis in Figure 5 indicate that $\tau_{D}^{SP}$ is the only model parameter that is uninfluential, since the sensitivity coefficients for all three RR series characteristics are below the dummy threshold. One important outcome of the sensitivity analysis therefore is that the refractory period and conduction delay of the AV node as well as the atrial input are influencing the RR series characteristics. For simplicity, we are proposing a linear scaling of refractory period and conduction delay parameters, but it would be interesting to refine this model description in the light of additional clinical data. It should be noted that the sensitivity coefficients $S_{n,m}$ are quantifying sensitivity on a global scale, and that there may be large local variations. As a result, the extent of variation in $\bar{RR}\left(t\right)$ , *RR*_*V*_(*t*) and *RR*_*I*_(*t*) for a set of scaling factors *A*_*R*_ and *A*_*D*_ depend on the model parameters. For example, in Figure 7, it is clear that the scaling factor *A*_*D*_ affects $\bar{RR}\left(t\right)$ , while the sensitivity analysis (Figure 5) indicates that the influence of changes in conduction delay on $\bar{RR}\left(t\right)$ is very limited on a global scale.

In the present study, the estimates in $RR_{V}^{E}\left(t\right)$ and $RR_{I}^{E}\;\left(t\right)$ were based on sliding windows of *N* = 200 RR intervals. The choice of *N* is a tradeoff between estimation accuracy and time resolution. The sample entropy estimation is expected to stabilize with greater *N* and a minimum of *N* ≥ 200 was recommended by Yentes et al. (2013). In the present study, *N* was chosen as short as possible in favour of time resolution to investigate the ANS-induced changes in the RR series characteristics during tilt. To accommodate the estimation uncertainty resulting from a small *N*, the simulated RR series characteristics trends were averaged over 10 repeated simulations for 240 different parameter sets. For the sensitivity analysis, *N* was chosen to be 4,000 in favour of estimation accuracy since the simulation was stationary.

While ANS modulation has been extensively studied during normal sinus rhythm (Porta et al., 2007; Porta et al., 2015; Sassi et al., 2015; Patel et al., 2018), no attempts have been made towards the estimation of ANS modulation during persistent AF. The present study is a first step towards developing a model of the AV node that will ultimately be used to quantify ANS modulation on a patient specific basis by fitting to RR interval series and information on atrial electrical activity obtained from clinical ECG recordings. The results (Figure 7) show that the proposed extended model of the AV node accounting for changes in autonomic tone can better replicate changes in RR series characteristics observed during tilt-test than the original model, implying that this is a viable approach to take. Further developments are needed to incorporate ANS modulation in the model and methodology for robust estimation of such modulation from clinical data.

## 5 Conclusion

We present an extended AV node model that incorporates ANS-induced changes. The extension was guided by a distribution-based sensitivity analysis showing that changes in refractoriness and conduction delay of the AV node as well as changes in atrial activity significantly influence the RR series characteristics. We demonstrate that the model extension is needed to replicate the changes in heart rate and RR series variability and irregularity observed during head-up and head-down tilt.

## Data Availability

The data analyzed in this study is subject to the following licenses/restrictions: The data is owned by the Department of Cardiology, Clinical Sciences, Lund University, Sweden. Requests to access these datasets should be directed to pyotr.platonov@ med.lu.se. The code for the extended model together with a user example can be found in the Supplementary Material.

## References

[B1] Bertrix L. Bouzouita K. Lang J. Lakhal M. Chah Q. T. Faucon G. (1984). Potentiation by hypokalemia of the effects of acetylcholine on the canine heart *in situ* . Naunyn. Schmiedeb. Arch. Pharmacol. 326, 169–174. 10.1007/BF00517315 6472494

[B2] Billette J. Tadros R. (2019). An integrated overview of av node physiology. Pacing Clin. Electrophysiol. 42, 805–820. 10.1111/pace.13734 31144331

[B3] Climent A. M. Atienza F. Millet J. Guillem M. S. (2011a). Generation of realistic atrial to atrial interval series during atrial fibrillation. Med. Biol. Eng. Comput. 49, 1261–1268. 10.1007/s11517-011-0823-2 21830052

[B4] Climent A. M. Guillem M. S. Zhang Y. Millet J. Mazgalev T. N. (2011b). Functional mathematical model of dual pathway av nodal conduction. Am. J. Physiol. Heart Circ. Physiol. 300, 1393–1401. 10.1152/ajpheart.01175.2010 21257912

[B5] Cohen R. J. Berger R. D. Dushane T. E. (1983). A quantitative model for the ventricular response during atrial fibrillation. IEEE Trans. Biomed. Eng. 30, 769–781. 10.1109/TBME.1983.325077 6662535

[B6] Corino V. D. Sandberg F. Mainardi L. T. Sörnmo L. (2011). An atrioventricular node model for analysis of the ventricular response during atrial fibrillation. IEEE Trans. Biomed. Eng. 58, 3386–3395. 10.1109/TBME.2011.2166262 21878405

[B7] Corino V. D. Ulimoen S. R. Enger S. Mainardi L. T. Tveit A. Platonov P. G. (2015). Rate-control drugs affect variability and irregularity measures of RR intervals in patients with permanent atrial fibrillation. J. Cardiovasc. Electrophysiol. 26, 137–141. 10.1111/jce.12580 25367150

[B8] Cossú S. F. Rothman S. A. Chmielewski I. L. Hsia H. H. Vogel R. L. Miller J. M. (1997). The effects of isoproterenol on the cardiac conduction system: Site-specific dose dependence. J. Cardiovasc. Electrophysiol. 8, 847–853. 10.1111/j.1540-8167.1997.tb00845.x 9261710

[B9] Cranefield P. F. Hoffman B. F. Paes de Carvalho A. (1959). Effects of acetylcholine on single fibers of the atrioventricular node. Circ. Res. 7, 19–23. 10.1161/01.RES.7.1.19 13619037

[B10] Denes P. Wu D. Dhingra R. Pietras R. J. Rosen K. M. (1974). The effects of cycle length on cardiac refractory periods in man. Circulation 49, 32–41. 10.1161/01.CIR.49.1.32 4271710

[B11] Dhingra R. C. Winslow E. Pouget J. M. Rahimtoola S. H. Rosen K. M. (1973). The effect of isoproterenol on atrioventricular and intraventricular conduction. Am. J. Cardiol. 32, 629–636. 10.1016/S0002-9149(73)80055-4 4744693

[B12] George S. A. Faye N. R. Murillo-Berlioz A. Lee K. B. Trachiotis G. D. Efimov I. R. (2017). At the atrioventricular crossroads: Dual pathway electrophysiology in the atrioventricular node and its underlying heterogeneities. Arrhythm. Electrophysiol. Rev. 6, 179–185. 10.15420/aer.2017.30.1 29326832 PMC5739891

[B13] Hageman G. R. Neely B. H. Urthaler F. James T. N. (1985). Negative chronotropic and parasympatholytic effects of alinidine on canine sinus node and AV junction. Am. J. Physiol. 248, 324–330. 10.1152/ajpheart.1985.248.3.H324 3976903

[B14] Hashimoto T. Fukatani M. Mori M. Hashiba K. (1991). Effects of standing on the induction of paroxysmal supraventricular tachycardia. J. Am. Coll. Cardiol. 17, 690–695. 10.1016/S0735-1097(10)80185-8 1993789

[B15] Henriksson M. Corino V. D. Sörnmo L. Sandberg F. (2016). A statistical atrioventricular node model accounting for pathway switching during atrial fibrillation. IEEE Trans. Biomed. Eng. 63, 1842–1849. 10.1109/TBME.2015.2503562 26625403

[B16] Henriksson M. Marozas V. Sandberg F. Sörnmo L. (2018). PetrnasModel-based assessment of f-wave signal quality in patients with atrial fibrillation. IEEE Trans. Biomed. Eng. 65, 2600–2611. 10.1109/TBME.2018.2810508 29993509

[B17] Hindricks G. Potpara T. Dagres N. Arbelo E. Bax J. J. Blomström-Lundqvist C. (2020). 2020 ESC Guidelines for the diagnosis and management of atrial fibrillation developed in collaboration with the European Association for Cardio-Thoracic Surgery (EACTS): The Task Force for the diagnosis and management of atrial fibrillation of the European Society of Cardiology (ESC) Developed with the special contribution of the European Heart Rhythm Association (EHRA) of the ESC. Eur. Heart J. 42, 373–498. 10.1093/eurheartj/ehaa612 32860505

[B18] Inada S. Shibata N. Iwata M. Haraguchi R. Ashihara T. Ikeda T. (2017). Simulation of ventricular rate control during atrial fibrillation using ionic channel blockers. J. Arrhythm. 33, 302–309. 10.1016/j.joa.2016.12.002 28765761 PMC5529332

[B19] Jørgensen P. Schäfer C. Guerra P. G. Talajic M. Nattel S. Glass L. (2002). A mathematical model of human atrioventricular nodal function incorporating concealed conduction. Bull. Math. Biol. 64, 1083–1099. 10.1006/bulm.2002.0313 12508532

[B20] Karlsson M. Sandberg F. Ulimoen S. R. Wallman M. (2021). Non-invasive characterization of human AV-nodal conduction delay and refractory period during atrial fibrillation. Front. Physiol. 12, 728955. 10.3389/fphys.2021.728955 34777001 PMC8584495

[B21] Lian J. Müssig D. Lang V. (2006). Computer modeling of ventricular rhythm during atrial fibrillation and ventricular pacing. IEEE Trans. Biomed. Eng. 53, 1512–1520. 10.1109/TBME.2006.876627 16916085

[B22] Lister J. W. Stein E. Kosowsky B. D. Lau S. H. Damato A. N. (1965). Atrioventricular conduction in man: Effect of rate, exercise, isoproterenol and atropine on the p-r interval. Am. J. Cardiol. 16, 516–523. 10.1016/0002-9149(65)90028-7 5834472

[B23] Lombardi F. Tarricone D. Tundo F. Colombo F. Belletti S. Fiorentini C. (2004). Autonomic nervous system and paroxysmal atrial fibrillation: A study based on the analysis of RR interval changes before, during and after paroxysmal atrial fibrillation. Eur. Heart J. 25, 1242–1248. 10.1016/j.ehj.2004.05.016 15246643

[B24] Mangin L. Vinet A. Pagé P. Glass L. (2005). Effects of antiarrhythmic drug therapy on atrioventricular nodal function during atrial fibrillation in humans. Europace 7, S71–S82. 10.1016/j.eupc.2005.03.016 16102505

[B25] Martin P. (1975). Dynamic vagal control of atrial-ventricular condition: Theoretical and experimental studies. Ann. Biomed. Eng. 3, 275–295. 10.1007/BF02390973 1220583

[B26] Martin P. (1977). The influence of the parasympathetic nervous system on atrioventricular conduction. Circ. Res. 41, 593–599. 10.1161/01.RES.41.5.593 332404

[B27] Morady F. Nelson S. D. Kou W. H. Pratley R. Schmaltz S. De Buitleir M. (1988). Electrophysiologic effects of epinephrine in humans. J. Am. Coll. Cardiol. 11, 1235–1244. 10.1016/0735-1097(88)90287-2 2835408

[B28] Nagaya K. Wada F. Nakamitsu S. Sagawa S. Shiraki K. (1995). Responses of the circulatory system and muscle sympathetic nerve activity to head-down tilt in humans. Am. J. Physiol. 268, R1289–R1294. 10.1152/ajpregu.1995.268.5.R1289 7771592

[B29] Östenson S. Corino V. D. Carlsson J. Platonov P. G. (2017). Autonomic influence on atrial fibrillatory process: Head-up and head-down tilting. Ann. Noninvasive Electrocardiol. 22, e12405. 10.1111/anec.12405 PMC6931695 27611110

[B30] Patel H. C. Hayward C. Wardle A. J. Middleton L. Lyon A. R. Di Mario C. (2018). The effect of head-up tilt upon markers of heart rate variability in patients with atrial fibrillation. Ann. Noninvasive Electrocardiol. 23, e12511. 10.1111/anec.12511 29034583 PMC6931507

[B31] Pianosi F. Wagener T. (2018). Distribution-based sensitivity analysis from a generic input-output sample. Environ. Model. Softw. 108, 197–207. 10.1016/j.envsoft.2018.07.019

[B32] Pirola F. T. Potter E. K. (1990). Vagal action on atrioventricular conduction and its inhibition by sympathetic stimulation and neuropeptide y in anaesthetised dogs. J. Auton. Nerv. Syst. 31, 1–12. 10.1016/0165-1838(90)90166-g 2262662

[B33] Porta A. Gnecchi-Ruscone T. Tobaldini E. Guzzetti S. Furlan R. Montano N. (2007). Progressive decrease of heart period variability entropy-based complexity during graded head-up tilt. J. Appl. Physiol. 103, 1143–1149. 10.1152/japplphysiol.00293.2007 17569773

[B34] Porta A. Faes L. Marchi A. Bari V. De Maria B. Guzzetti S. (2015). Disentangling cardiovascular control mechanisms during head-down tilt via joint transfer entropy and self-entropy decompositions. Front. Physiol. 6, 301. 10.3389/fphys.2015.00301 26578973 PMC4621422

[B35] Priola D. V. Curtis M. B. Anagnostelis C. Martinez E. (1983). Altered nicotinic sensitivity of AV node in surgically denervated canine hearts. Am. J. Physiol. 245, 27–32. 10.1152/ajpheart.1983.245.1.H27 6869561

[B36] Rashidi A. Khodarahmi I. (2005). Nonlinear modeling of the atrioventricular node physiology in atrial fibrillation. J. Theor. Biol. 232, 545–549. 10.1016/j.jtbi.2004.08.033 15588634

[B37] Richman J. S. Moorman J. R. (2000). Physiological time-series analysis using approximate entropy and sample entropy. Am. J. Physiol. Heart Circ. Physiol. 278, 2039–2049. 10.1152/ajpheart.2000.278.6.H2039 10843903

[B38] Sassi R. Cerutti S. Lombardi F. Malik M. Huikuri H. V. Peng C.-K. (2015). Advances in heart rate variability signal analysis: Joint position statement by the e-cardiology ESC working group and the European heart rhythm association co-endorsed by the asia pacific heart rhythm society. Europace 17, 1341–1353. 10.1093/europace/euv015 26177817

[B39] Shen M. J. Zipes D. P. (2014). Role of the autonomic nervous system in modulating cardiac arrhythmias. Circ. Res. 114, 1004–1021. 10.1161/CIRCRESAHA.113.302549 24625726

[B40] Spear J. F. Moore E. N. (1973). Influence of brief vagal and stellate nerve stimulation on pacemaker activity and conduction within the atrioventricular conduction system of the dog. Circ. Res. 32, 27–41. 10.1161/01.RES.32.1.27 4684126

[B41] Trautwein W. (1963). Generation and conduction of impulses in the heart as affected by drugs. Pharmacol. Rev. 15, 277–332. 13994011

[B42] Wallman M. Sandberg F. (2018). Characterisation of human AV-nodal properties using a network model. Med. Biol. Eng. Comput. 56, 247–259. 10.1007/s11517-017-1684-0 28702812

[B43] West T. C. Toda N. (1967). Response of the A-V node of the rabbit to stimulation of intracardiac cholinergic nerves. Circ. Res. 20, 18–31. 10.1161/01.res.20.1.18 6018221

[B44] Yentes J. M. Hunt N. Schmid K. K. Kaipust J. P. McGrath D. Stergiou N. (2013). The appropriate use of approximate entropy and sample entropy with short data sets. Ann. Biomed. Eng. 41, 349–365. 10.1007/s10439-012-0668-3 23064819 PMC6549512

