# Supplementary material for: An atrioventricular node model incorporating autonomic tone
Source: Front Physiol. 2022 Sep 15;13:976468. doi: 10.3389/fphys.2022.976468 (PMC9520409; doi:10.3389/fphys.2022.976468)
Supplement: Supplementary file 1 [file DataSheet1.PDF]

Supplementary Material

1 PARAMETER SET SELECTION

The same parameter set generation and exclusion criteria as in Sec. 2.4 were applied to obtain the parameter sets used for the simulations in Sec. 2.6. In addition to the exclusion criteria from Sec. 2.4, parameter sets were also excluded if the average refractory period of both SP and FP were above 600 ms and the average AH interval was below 100 ms or above 500 ms. The average refractory period of a pathway was computed as the average of all computed refractory period values for all nodes in a pathway and all impulses that resulted in a ventricular activation. The average AH interval was computed as the average of all time differences between atrial and ventricular activation for all impulses that resulted in a ventricular activation. Instead of simulating 4000 RR intervals with fixed μ and σ as in Sec. 2.4, the RR interval series are simulated with AA series generated from the population-averaged $\mu(t)$ and $\sigma(t)$ during supine position. To obtain more robust estimates for the parameter set selection, the simulations for each parameter set were repeated 10 times, while the $\overline{RR}$ and RR_V were computed from the entire RR series resulting from each realization. The resulting $\overline{RR}$, RR_V as well as the average refractory periods and average AH interval were averaged. Both the clinical and simulated RR series characteristics were sorted by their $\overline{RR}$ and RR_V values according to a 20×20 ms grid. For each clinical RR series, 10 model parameter sets were randomly drawn from the same grid field. The resulting parameters are displayed in Table S1 and summarized in Table 2.

Table S1. Parameters used for the simulations in Sec. 2.6 (mean±std).

Patient	R^{SP}_{min}	ΔR^{SP}	τ^{SP}_{R}	D^{SP}_{min}	ΔD^{SP}	τ^{SP}_{D}	R^{FP}_{min}	ΔR^{FP}	τ^{FP}_{R}	D^{FP}_{min}	ΔD^{FP}	τ^{FP}_{D}
1	361 ± 81	219 ± 91	132 ± 71	18 ± 10	41 ± 18	219 ± 36	478 ± 71	408 ± 140	170 ± 70	5 ± 8	28 ± 23	173 ± 86
2	344 ± 72	212 ± 66	147 ± 77	19 ± 5	46 ± 20	138 ± 84	514 ± 86	419 ± 135	152 ± 66	7 ± 4	20 ± 14	162 ± 68
3	302 ± 41	243 ± 125	156 ± 90	19 ± 8	46 ± 14	154 ± 79	473 ± 99	371 ± 92	195 ± 68	6 ± 8	29 ± 24	149 ± 78
4	319 ± 76	262 ± 127	191 ± 51	21 ± 9	41 ± 16	127 ± 67	504 ± 53	378 ± 176	203 ± 62	5 ± 9	22 ± 15	169 ± 68
5	331 ± 50	214 ± 99	138 ± 75	19 ± 8	46 ± 21	183 ± 88	469 ± 96	420 ± 155	174 ± 69	9 ± 7	23 ± 20	139 ± 69
6	300 ± 40	285 ± 54	114 ± 59	21 ± 6	42 ± 21	189 ± 84	446 ± 112	393 ± 139	132 ± 59	7 ± 6	29 ± 17	174 ± 68
7	341 ± 72	239 ± 139	167 ± 81	21 ± 7	34 ± 14	171 ± 88	503 ± 100	323 ± 214	126 ± 74	10 ± 7	20 ± 10	127 ± 47
8	318 ± 66	241 ± 129	170 ± 62	21 ± 8	48 ± 19	172 ± 72	495 ± 99	362 ± 226	181 ± 62	6 ± 8	34 ± 26	150 ± 82
9	336 ± 74	208 ± 110	174 ± 95	19 ± 8	39 ± 25	158 ± 69	506 ± 68	387 ± 172	158 ± 79	11 ± 7	21 ± 10	161 ± 80
10	345 ± 96	282 ± 158	185 ± 83	23 ± 6	37 ± 23	169 ± 49	478 ± 94	374 ± 122	181 ± 94	8 ± 6	18 ± 18	146 ± 73
11	305 ± 68	269 ± 77	153 ± 84	17 ± 7	33 ± 20	160 ± 85	484 ± 93	385 ± 135	146 ± 63	8 ± 8	21 ± 16	182 ± 79
12	320 ± 90	227 ± 101	124 ± 52	19 ± 7	41 ± 20	167 ± 84	526 ± 99	311 ± 176	209 ± 71	9 ± 7	18 ± 19	163 ± 70
13	345 ± 91	224 ± 112	158 ± 85	18 ± 4	46 ± 24	170 ± 77	462 ± 88	388 ± 153	118 ± 77	5 ± 5	20 ± 17	142 ± 63
14	337 ± 85	252 ± 125	124 ± 80	21 ± 5	39 ± 23	199 ± 84	504 ± 55	464 ± 155	154 ± 51	5 ± 5	14 ± 12	176 ± 65
15	318 ± 77	269 ± 103	185 ± 93	19 ± 9	35 ± 25	193 ± 73	464 ± 83	456 ± 94	160 ± 73	9 ± 11	11 ± 6	143 ± 76
16	345 ± 72	208 ± 89	189 ± 81	21 ± 6	34 ± 26	163 ± 36	489 ± 86	349 ± 135	183 ± 84	4 ± 3	32 ± 24	128 ± 68
17	360 ± 68	189 ± 98	156 ± 56	22 ± 7	39 ± 14	190 ± 49	479 ± 71	320 ± 179	164 ± 68	6 ± 4	20 ± 20	200 ± 78
18	342 ± 60	233 ± 111	157 ± 85	23 ± 5	24 ± 21	142 ± 52	475 ± 69	429 ± 120	162 ± 58	8 ± 4	14 ± 13	162 ± 88
19	351 ± 73	228 ± 125	191 ± 82	16 ± 7	35 ± 21	185 ± 44	527 ± 58	321 ± 207	160 ± 73	8 ± 6	22 ± 21	152 ± 60
20	323 ± 70	236 ± 176	148 ± 97	22 ± 7	31 ± 23	138 ± 57	533 ± 40	465 ± 186	193 ± 91	6 ± 4	14 ± 6	136 ± 71
21	302 ± 50	224 ± 104	151 ± 54	21 ± 8	40 ± 21	152 ± 86	453 ± 65	420 ± 161	131 ± 66	8 ± 7	24 ± 7	142 ± 64
22	313 ± 71	269 ± 82	169 ± 75	18 ± 7	40 ± 22	164 ± 52	455 ± 88	423 ± 122	139 ± 71	6 ± 3	20 ± 10	159 ± 70
23	319 ± 60	307 ± 75	209 ± 75	20 ± 8	35 ± 27	180 ± 73	480 ± 104	403 ± 145	163 ± 92	10 ± 6	20 ± 18	145 ± 56
24	372 ± 82	167 ± 60	124 ± 43	21 ± 5	38 ± 21	141 ± 65	493 ± 92	350 ± 146	145 ± 66	7 ± 4	10 ± 9	171 ± 95
